# Single-cell transcriptomics identify a novel macrophage population associated with bone invasion in pituitary neuroendocrine tumors

**DOI:** 10.1186/s13046-025-03296-9

**Published:** 2025-01-27

**Authors:** Xinzhi Wu, Xueshuai Han, Haibo Zhu, Mingxuan Li, Lei Gong, Sicheng Jing, Weiyan Xie, Zhaoqi Liu, Chuzhong Li, Yazhuo Zhang

**Affiliations:** 1 Beijing Neurosurgical Institute, Capital Medical University, Beijing, 100070 China; 2 Beijing Institute of Genomics, China National Center for Bioinformation, Chinese Academy of Sciences, Beijing, 100101 China; 3 Department of Neurosurgery, Beijing Tiantan Hospital Affiliated to Capital Medical University, Beijing, 100070 China; 4 Department of Biology, University of California San Diego, San Diego, CA 92122 USA

**Keywords:** PitNETs, Bone invasion, TAM, TNF-α, Macrophage

## Abstract

**Background:**

Bone-invasive Pituitary Neuroendocrine Tumors (BI PitNETs) epitomize an aggressive subtype of pituitary tumors characterized by bone invasion, culminating in extensive skull base bone destruction and fragmentation. This infiltration poses a significant surgical risk due to potential damage to vital nerves and arteries. However, the mechanisms underlying bone invasion caused by PitNETs remain elusive, and effective interventions for PitNET-induced bone invasion are lacking in clinical practice.

**Methods:**

In this study, we performed single-cell (*n* = 87,287) RNA sequencing on 10 cases of bone-invasive PitNETs and 5 cases of non-bone-invasion PitNETs (Non-BI PitNETs). We identified various cell types and determined their interactions through cell-cell communication analysis, which was further validated experimentally.

**Results:**

We identified a novel TNF-α^+^ TAM macrophage subset. BI PitNETs showed increased IL-34 secretion, impacting TNF-α^+^ TAMs via the IL34/CSF1R axis, leading to TNF-α production. TNF-α^+^ TAMs, in turn, communicate with *CD14*^+^ monocytes to promote their differentiation into osteoclasts and leading to bone invasion. In addition, we defined a gene signature for TNF-α^+^ TAM to guide the clinical prognosis prediction of BI PitNETs.

**Conclusions:**

Our study elucidates the tumor microenvironment changes in bone invasion and identifies the critical role of TNF-α^+^ TAMs in promoting bone invasion of PitNETs, laying a foundation for developing new molecular markers or therapeutic agents targeting BI PitNETs.

**Supplementary Information:**

The online version contains supplementary material available at 10.1186/s13046-025-03296-9.

## Background

Pituitary neuroendocrine tumors (PitNETs) are prevalent neoplasms originating from the anterior pituitary gland, comprising approximately 17.1% of intracranial tumors [1]. PitNETs may lead to clinical symptoms either excessive hormone secretion or due to the mass effect of the tumor. Although most PitNETs are benign, a subset demonstrates aggressive behavior by infiltrating the surrounding tissues and therefore, posing challenges during surgical removal, decreasing the chance of complete resection, and increasing the risk of recurrence. Among them, bone-invasive PitNETs (BI PitNETs) are the most aggressive type. Normal bone invaded by the tumor often becomes fragmented and enveloped by tumor tissue, necessitating delicate intraoperative handling. Improper management during surgery can result in direct damage to vital blood vessels, cranial nerves, dura mater, or brain tissue, markedly increasing surgical complexity and risks [2]. Currently, effective treatment modalities for such tumors remain elusive.

In recent years, research on the tumor microenvironment (TME) and immunotherapy has gained prominence in oncology [3]. Interactions among these components have been proven to promote tumor initiation, progression, invasion, and metastasis [4]. Studies have indicated that infiltration of various immune cells within the TME of PitNETs is highly correlated with tumor progression and invasion [5, 6]. Notably, macrophages are the most abundant immune cells in the PitNET TME and play pivotal roles [7]. While preliminary research on the impact of TME on bone invasion has been conducted in other tumors, the underlying mechanisms remain largely unknown. In addition, studies on the role of TME in PitNETs -induced bone invasion are still lacking.

Single-cell RNA sequencing (scRNA-seq) technology has been widely employed to investigate the composition of the TME and the associated tumor pathogenesis [8]. In this study, we performed single-cell (*n* = 87,287) RNA sequencing on 10 cases of bone-invasive PitNETs and 5 cases of non-bone-invasion PitNETs (Non-BI PitNETs). We established a cellular atlas of the immune microenvironment specific to bone-invasive PitNETs and identified a novel subset of macrophages. Through cell-cell communication analysis, we further elucidated their crucial role in the process of bone invasion, offering new perspectives for the treatment and prevention of bone invasion in patients with PitNETs.

## Materials and methods

### Patients and clinical specimens

From October 2022 to March 2023, we collected 10 BI and 5 Non-BI PitNET samples from patients undergoing tumor resection at Beijing Tiantan Hospital for single-cell sequencing, with sample details in Supplementary Table S1.

To determine whether different pathological types affect the invasiveness of PitNETs, we conducted a comparative analysis of the invasiveness of bone in different types of PitNETs using a group of 190 bulk sequencing samples. This confirmed that there is no significant correlation between bone invasion and pathological types. Detailed results can be found in Supplementary Table S2.

Therefore, in conducting this study, we mainly focused on the bone invasion status and did not differentiate pathological types during the grouping. One additional BI sample was collected for spatial transcriptomics. All diagnoses were confirmed by a multidisciplinary team. Bone-invasive PitNETs were classified based on MRI (Knosp grades 3–4, Hardy–Wilson grades 3–4, or stage D-E), CT (involvement of sellar floor, slopes, or discontinuous bone cortex), and intraoperative findings (bone invasion with fragments). Ethical approval was obtained (KY 2018-053-02), and informed consent was provided by all patients.

### Preprocessing of single-cell samples and spatial transcriptomics sample

Fresh PitNET tissues were digested with PBS, collagenase II/IV for 30 min at 37 °C, then filtered through a 45-µm mesh. After resuspension in L15 medium with 10% FBS, a second digestion with Accutase dissociated cell clusters. Immune cells were enriched using CD45 magnetic beads and mixed with CD45^−^ cells at a 1:1 ratio, then cultured in L15 medium with 10% FBS.

### Single-cell RNA sequencing

Single-cell suspensions were processed using the Chromium Single-Cell Expression Solution (10× Genomics) to capture 5000-10,000 cells per library. Libraries were sequenced on the HiSeq4000 platform (Illumina) with 150 bp paired-end reads.

### Bulk RNA-seq analysis

Reads were aligned to the human hg38 reference genome using the STAR aligner (v 2.7.7a) [9]. Read count matrices were generated using the featureCounts function from the Rsubread package (v 2.0.1). These matrices were finally converted to TPM values, and then log2 transformed [10].

### scRNA-seq data processing

Raw sequencing data were processed using Cell Ranger (v6.0.2) with default settings and aligned to GRCH38 to generate gene expression matrices. These were analyzed with Scanpy (v1.8.1) [11] for quality control and downstream analysis. Low-quality cells (fewer than 200 genes/cell and fewer than 3 cells/gene) and outliers (cells with > 6500 expressed genes or > 20% mitochondrial gene content) were excluded. Highly variable genes were identified, and batch effects were corrected with BBKNN. PCA, UMAP, and graph-based clustering were used for analysis.

### Differential expression and enrichment analysis

Marker genes for clusters were identified using Seurat (v4.3.0.1), with a P value < 0.05 (Wilcoxon test) as the threshold. GO and pathway enrichment analysis was done with Metascape [12] and GSEA (clusterProfiler v4.8.2) [13].

### Calculating gene set scores

The gene scores were computed using the AddModuleScoreUCell function from the UCell package [14], utilizing default parameters.

### RNA velocity and pseudotime analysis

RNA velocity was calculated via Velocyto (v0.17.17), and velocity fields were projected onto UMAP embeddings. Pseudotime relationships were determined using Monocle3 (v1.3.4) and PAGA.The Monocle3 package (v 1.3.4) [15] and PAGA [16]were used to determine the pseudotime developmental relationships of each cluster by using default parameters.

### Inferring CNVs from scRNA-seq data

The single-cell CNVs were identified using the InferCNV R package (v1.8.1) [17, 18], which estimates chromosomal variants by comparing gene expression across loci in tumor cells to normal reference cells. Kmeans clustering was used to distinguish normal cells within tumor samples, with normal pituitary cells (Normal1, Normal8, Normal25) serving as the reference. InferCNV was run separately for each tumor sample using the raw count matrix, filtering out genes with an average read count below 0.1 in the reference cells.

### Cell–cell communication analysis

We used CellChat (v 1.6.1) [19]based on the CellChatDB database to infer cell-cell interactions of selected ligand-receptor pairs between tumor cells and tumor microenvironment cell subpopulations. Cellular communication networks were inferred by identifying differentially overexpressed ligands and receptors in each cell population. The computeCommunProb function was utilized with parameters set to “truncatedMean” to compute the communication probability.

### Spatial transcriptomic data processing

Spatial transcriptomic data were aligned to their corresponding HE images using SpaceRanger (v2.1.0). Genes with fewer than 10 counts and spots with fewer than 10 genes were filtered out. Cell-type proportions were inferred using DestVI (v0.1.0) after training scLVM on scRNA-seq data, followed by stLVM training for spatial cell type extraction. Spatial patterns were analyzed using gamma space and Spatial PCs, and gene expression values were imputed from the trained scLVM with the get_scale_for_ct() function.

### Deconvolution of bulk RNA-seq data

The fraction of cell types in bulk RNA-seq samples was inferred using the CIBERSORTx online tool [20]. Single-cell reference data were curated by randomly selecting 100 cells per annotated cell type from our scRNA-seq dataset. Subsequently, we utilized the Create Signature Matrix function to create cell-type signature matrices, followed by the Impute Cell Fractions function to estimate the relative cell fractions in each tumor sample, employing default parameters throughout the process.

### Definition of TNF-α+ TAM gene signature

We performed differential gene expression analysis on TNF-α^+^ TAM from both the bone-invasive and non-bone-invasive groups, identifying genes with a log2FC ≥ 0.25 as TNF-α^+^ TAM-specific. Cox multivariate regression coefficients were employed to evaluate the prognostic impact of these genes. Ultimately, we identified four genes associated with high risk and with a P value < 0.05 as the TNF-α^+^ TAM gene signature.

### Survival analysis

The Kaplan-Meyer curve and log-rank test (Mantel-Cox test) p-values were used to quantify the difference in progression-free survival (PFS) between high-expression and low-expression groups. Survival analysis was performed using the survival R package (v 3.5.5, https://github.com/therneau/survival).

### Statistics and reproducibility

Statistical analyses were performed using R v4.3.1 or GraphPad Prism 9. Spearman’s correlation tested correlations, and the Wilcoxon rank sum test identified significant differences between groups. FDR was calculated using the Benjamin–Hochberg method. Significance levels were denoted as *p < 0.05, **p < 0.01, ***p < 0.001. Detailed descriptions of tests are provided in the figures and legends.

### RNA extraction and RT-qPCR

Total RNA was extracted from human PitNET tissues using the SteadyPure Quick RNA Extraction Kit and reverse transcribed with the biosharp Reverse Transcription Kit. qRT-PCR was performed with SYBR Green assays on the ABI 7500 System, using GAPDH as the internal control. Expression levels were calculated via the 2−∆∆CT method and normalized to GAPDH. All analyses were triplicated, with significance assessed by student’s t-tests (*P* < 0.05). Primer sequences are in Supplementary Table S3.

### Multiplexed immunohistochemistry

Multiplexed immunohistochemistry (mIHC) was performed by sequentially staining 4-um-thick formalin-fixed, paraffin-embedded whole tissue sections with standard primary antibodies and paired with TSA 5-color kit (abs50013-100T, Absinbio, Shanghai). The primary antibodies used included anti-CX3CR1/ab184678, anti-TREM2/ab85851, anti-CD68/absin171440, anti-TNF/ab6671, and anti-IL34/ab224734. After air-drying, images were captured with the Pannoramic MIDI II (3DHISTECH) and analyzed with Indica Halo software.

## Results

### The immune landscape of bone-invasive PitNETs

**Fig. 1:**
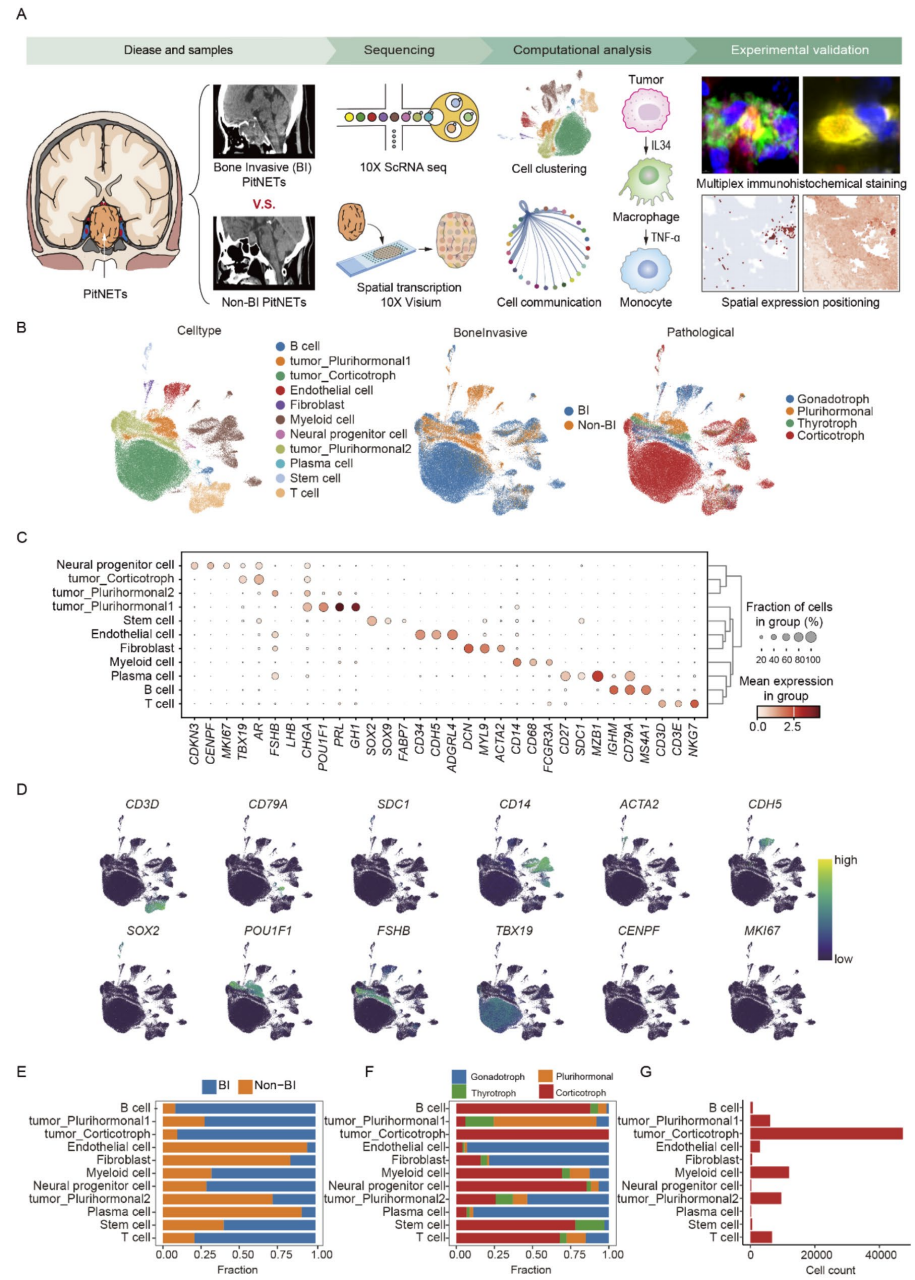
Single-cell atlas of Bone Invasive and Non-Bone Invasive PitNET. **A** Graphic overview of the study design. Tumor tissues derived from patients with Bone Invasive (BI) and Non-Bone Invasive (Non-BI) PitNETs were converted into single-cell suspensions. Unsorted cells were employed for single-cell RNA sequencing (scRNA-seq) using the 10XGenomics platform. Additionally, spatial transcriptomics data were acquired from tumor slides using the 10X Genomics Visium technology. Analysis revealed the presence of TNF-α^+^ macrophages in invasive bone PitNETs, implicating them in bone destruction. This finding was validated through spatial transcriptomics and multiple immunofluorescence staining. **B** Uniform manifold approximation and projection (UMAP) of all 87,287 cells from 10 BI (*n* = 65,171 cells) and 5 Non-BI patients (*n* = 22,116 cells), colored by assigned cell type, Bone Invasiveness and pathological condition. **C** Dot plot depicting the average expression of cell-type-specific markers in each cell cluster. The dot size represents the proportion of cells expressing each marker gene within each cluster. **D** UMAP plots showing the expression levels of selected marker genes. **E**, **F**, **G** Bar plots illustrating the proportion of each cell type across Bone Invasive (left panel) states and pathological conditions (middle panel), along with the total cell number for each cell type (right panel)

To delineate the immune landscape of PitNETs, we conducted single-cell transcriptome sequencing on 10 BI PitNETs and 5 Non-BI PitNETs (Fig. 1A). After quality control, 87,287 cells were included for further analysis, comprising 65,171 cells from BI samples and 22,116 cells from Non-BI samples. On average, these cells had 9,334 unique molecular identifiers (UMIs) and 2,799 expressed genes (Figure S1A). The 15 samples were categorized into four pathological types based on transcription factors (*POU1F1*, *TBX19*, and *NR5A1*) and hormone-secreting genes (*POMC*, *GH1*, *GH2*, *TSHB*, *PRL*, *FSHB*, and *LHB*). These types were named as Gonadotroph tumor, Plurihormonal tumor, Thyrotroph tumor, and Corticotroph tumor. Though unsupervised clustering, we identified 11 distinct cell types: B cells (marked by *IGHM*, *CD79A*, *MS4A1*), tumor_ Plurihormonal1 (*POU1F1*, *PRL*, *GH1*), tumor_ Corticotroph (*TBX19*, *AR*), endothelial cells (*CD34*, *CDH5*, *ADGRL4*), fibroblasts (*DCN*, *MYL9*, *ACTA2*), myeloid cells (*CD14*, *CD68*, *FCGR3A*), neural progenitor cells (*CDKN3*, *CENPF*), tumor_ Plurihormonal2 (*FSHB*, *LHB*, *CHGA*), plasma cells (*CD27*, *SDC1*, *MZB1*), stem cells (*SOX2*, *SOX9*, *FABP7*), and T cells (*CD3D*, *CD3E*, *NKG7*) (Fig. 1B, C). Previously defined marker genes consistently displayed distinct expression patterns unique to each corresponding subtype (Fig. 1D). Further analysis revealed that each cell type was derived from all 15 samples (Figure S1 B and C), and the cellular composition was generally consistent between BI and Non-BI samples, without notable donor-specific subgroups or batch effects. To assess the relationship between bone invasion and the proportions of different cell types, we compared the cell proportions from the BI and Non-BI groups within each cell type (Fig. 1E). Most cell types exhibited the highest proportion of cells in the BI group, which could be partially attributed to the relatively larger sample size. Interestingly, the Non-BI group showed a higher proportion of several cell types, including endothelial cells, fibroblasts, and plasma cells, which indicates that these cell types may play an important role in bone invasion. To further investigate the association between bone invasion and pathological classification, we analyzed the pathological origin of each cell type. Different types of tumor cells originate solely from their corresponding pathological types, whereas immune cells, fibroblasts, and endothelial cells originate from all pathological types (Fig. 1F). These findings highlight significant heterogeneity among tumor cells, while the non-tumor cells within the tumor microenvironments of different pathological classifications exhibit relatively less heterogeneity. Comparing the relative abundance of different cells, we found that tumor cells constituted the majority, followed by myeloid cells, T cells, fibroblasts, and B cells (Fig. 1G). To determine the presence of gene copy number variations in PitNETs, we performed inferCNV analysis, which accurately distinguished between tumor and non-tumor cells. The analysis revealed significant copy number variations in PitNETs tumor cells, whereas no variations were observed in non-tumor cells, indicating that non-tumor cells, primarily immune cells, are in a normal functional state (Figure S1 D and 1E).

### Two distinct states of tumor associated macrophages (TAMs) in PitNETs

**Fig. 2:**
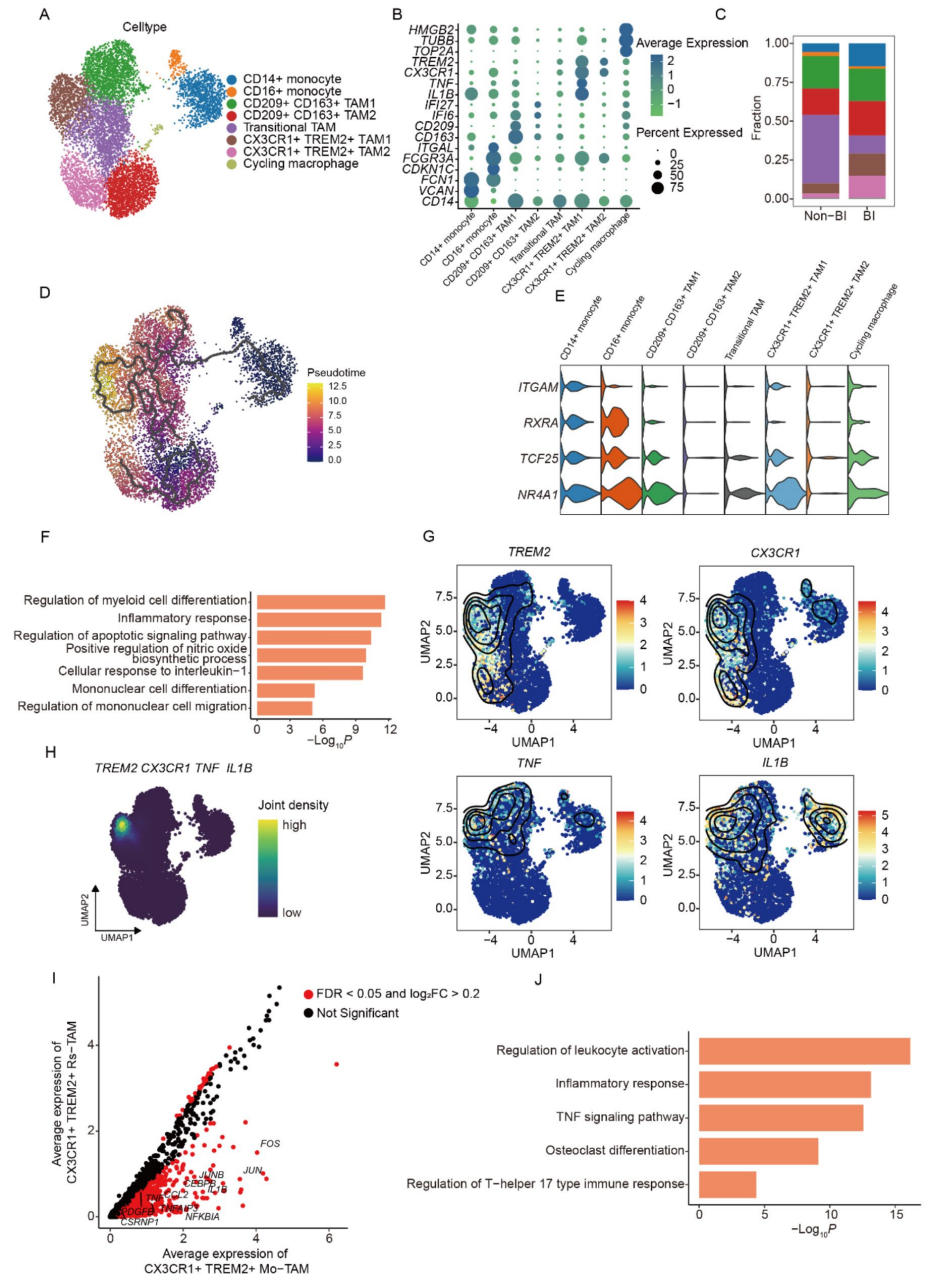
Two distinct states of tumor associated macrophages (TAMs) in PitNETs. **A** UMAP of 8,560 monocytes and macrophages colored by assigned cell type. **B** Dot plot depicting the average expression of cell-type-specific markers across cell clusters. The dot size indicates the proportion of cells expressing each marker gene within the cluster. **C** Bar plot displaying the proportion of each cell type across Bone Invasive states. **D** Inferred developmental trajectory of monocytes and macrophages using monocle3, with colors representing psedotime. **E** Violin plot showing the expression of myeloid-derived suppressor cells (MDSCs) signatures. **F** Bar plot depicting pathways enriched in genes that exhibit higher expression in monocyte-derived (Mo) macrophages compared to tissue-resident (Rs) macrophages. **G** UMAP plots showing the expression levels of selected marker genes in CX3CR1 + TREM2 + TAMs. **H** Density plot visualizing combined gene expression of CX3CR1 + TREM2 + Mo-TAMs markers. **I**, **J** Differentially expressed genes (left) and differentially activated pathways (right) between CX3CR1 + TREM2 + Mo-TAMs and CX3CR1 + TREM2 + Rs-TAMs

Next, to investigate the role of various myeloid cells in bone invasion, we isolated 10,346 myeloid cells for in-depth analysis. These cells were categorized based on distinct expression patterns of various cellular marker genes, resulting in the identification of eight myeloid subpopulations: *CD14*^+^ monocytes, *CD16*^+^ monocytes, macrophages, mast cells, *CLEC4C*^+^ plasmacytoid dendritic cells (pDCs), *CD1C*^+^ conventional dendritic cells (cDCs), neutrophils, and cycling macrophages (Supplementary Figure S2 A, B). Upon comparing the proportions of various cell types between the BI and Non-BI groups and categorizing all cells based on sample pathology, no significant differences were found between the groups (Supplementary Figure S2 C, D). Focusing on the most abundant cell types, monocytes and macrophages, which comprised 8,560 cells, further analysis delineated eight distinct cellular subtypes (Fig. 2A, B). Despite utilizing established molecular signatures for M1 and M2 macrophages, angiogenesis, and phagocytosis, these signatures proved insufficient in accurately discriminating between M1 and M2 macrophage phenotypes (Supplementary Figure S2E). Notably, macrophages were mainly divided into four subpopulations. Two of these subpopulations were characterized by high expression of *CX3CR1* and *TREM2*, which we designated as *CX3CR1*^+^*TREM2*^+^ TAM1 and *CX3CR1*^+^*TREM2*^+^ TAM2. Another subpopulation was characterized by high expression of *CD209* and *CD163*, designated as *CD209*^+^*CD163*^+^ TAM1 and *CD209*^+^*CD163*^+^ TAM2 (Fig. 2A, B). The proportions of *CX3CR1*^+^*TREM2*^+^ TAM1 and *CX3CR1*^+^*TREM2*^+^ TAM2 in the BI group were significantly higher than in the Non-BI group, suggesting a specific role in bone invasion (Fig. 2C). To investigate the differentiation origins of these four macrophage subpopulations, we conducted a pseudotime trajectory analysis and found that the differentiation origins of *CX3CR1*^+^*TREM2*^+^ TAM1 and *CX3CR1*^+^*TREM2*^+^ TAM2 are different (Fig. 2D). Therefore, we further used myeloid-derived suppressor cell (MDSC) markers to verify their differentiation origins and found that *CX3CR1*^+^*TREM2*^+^ TAM1 highly expressed MDSC markers, suggesting that they may originate from circulating monocytes. In contrast, *CX3CR1*^+^*TREM2*^+^TAM2 showed significantly low expression of MDSC markers, indicating that it may originate from tissue-resident macrophages (Fig. 2E). Furthermore, we performed enrichment analysis of differentially expressed genes to reveal the functional differences between monocyte-derived macrophages (Mo) and tissue-resident macrophages (Rs) (Supplementary Table S3). We found that upregulated genes in Mo were primarily enriched in pathways related to myeloid cell differentiation and inflammatory responses, indicating that they are in a different functional state (Fig. 2F). Previous studies have shown that TNF-α and IL-1B act as key molecules when promoting bone invasion of PitNETs by stimulating osteoclast differentiation. To determine whether *CX3CR1*^+^*TREM2*^+^ TAM1 can promote bone invasion through these two key molecules, we examined the expression patterns of *TNF* and *IL-1B*. Our analysis revealed that *CX3CR1*^+^*TREM2*^+^ TAM1 demonstrated markedly elevated expression levels of *TNF* and *IL1B* (Fig. 2G, H). Moreover, a comparative gene expression analysis between *CX3CR1*^+^*TREM2*^+^ Mo-TAM1 and *CX3CR1*^+^*TREM2*^+^ Rs-TAM2 highlighted significantly enriched pathways associated with leukocyte activation, inflammatory responses, TNF signaling, and osteoclast differentiation (Fig. 2I, J). Interestingly, by comparing the average expression levels of these differential genes, we found that the global expression levels in *CX3CR1*^+^*TREM2*^+^ Mo-TAM1 were higher than those in *CX3CR1*^+^*TREM2*^+^ Rs-TAM2 (Fig. 2I), suggesting that *CX3CR1*^+^*TREM2*^+^ Mo-TAM1 may be in a functionally active state. On the contrary, *CX3CR1*^+^*TREM2*^+^ TAM2 is in a functionally quiescent state. This suggests a distinct functional orientation of the *CX3CR1*^+^*TREM2*^+^ Mo-TAM1 phenotype (Abbreviated as TNF-α^+^TAM.), particularly concerning immune modulation and bone metabolism, which is integral for understanding the underlying mechanisms in pathological conditions. The proportion of *CD209*^+^*CD163*^+^ TAMs in the BI and Non-BI groups did not show a significant difference. However, they highly expressed *AHR* and *VCAM1*, suggesting their crucial roles in immune cell differentiation, cytokine expression, and cell adhesion processes (Figure S2 F). The differentially expressed genes between monocyte-derived and tissue-resident *CD209*^+^*CD163*^+^ TAMs were primarily enriched in pathways related to lymphocyte proliferation regulation (Figure S2 G, H). This further indicates that the functions of *CD209*^+^*CD163*^+^ TAMs are likely associated with the regulation of lymphocyte proliferation and differentiation, cytokine expression, and cell adhesion processes. In summary, we discovered a novel macrophage subset in the immune microenvironment of PitNETs, which we have named *CX3CR1*^+^*TREM2*^+^ TAM1(Abbreviated as TNF-α^+^TAM). This subset is significantly enriched in the immune microenvironment of bone-invasive PitNETs and exhibits high expression of TNF-α and IL1B. The TNF-α^+^TAM may be functionally related to lymphocyte activation, inflammatory response, TNF signaling pathway, and osteoclast differentiation, potentially playing a vital role in the bone invasion of PitNETs.

### Presentation of TNF-α+TAM in PitNETs is associated with increased TNF-α expression

**Fig. 3:**
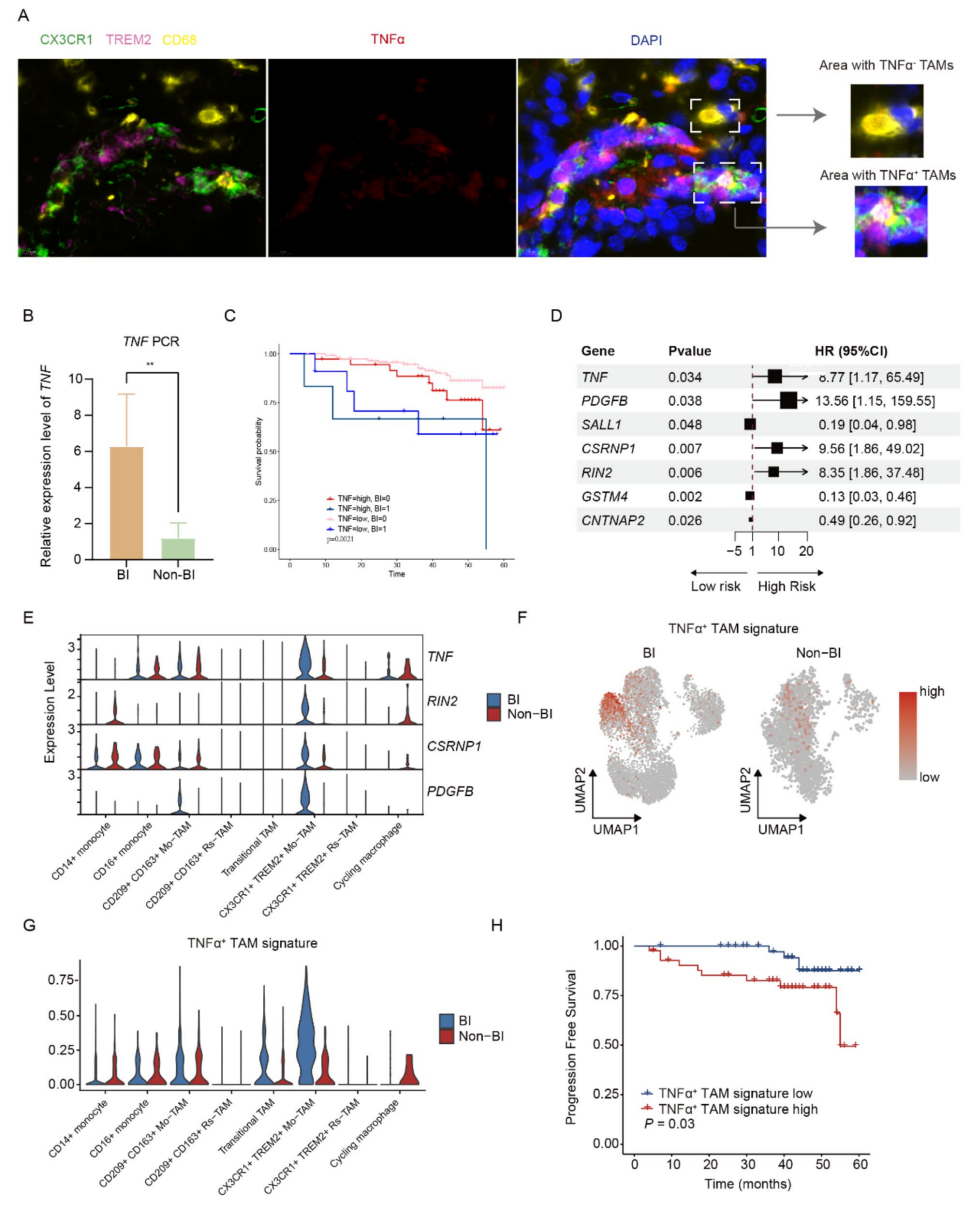
Identification of TNFα^+^ TAMs in PitNETs. **A** Multiple immunofluorescence staining reveals the expression of CX3CR1 (green), TREM2 (pink), CD68 (yellow), TNF-α (red), DAPI (blue), etc. in BI PitNET; TNF-α^+^ macrophages (CX3CR1^+^, TREM2^+^, CD68^+^, TNF-α^+^) and TNF-α^−^ macrophages (CX3CR1^−^, TREM2^−^, CD68^+^, TNF-α^−^) are observed. **B** PCR analysis detected the relative expression levels of TNF in 6 cases of BI and 5 cases of Non-BI PitNETs, showing significantly higher TNF expression in the BI group. **C** Kaplan-Meier plot displaying the Progression Free Survival in patients with PitNETs, stratified by TNF expression levels and bone invasion status at the first quartile cutoff point. **D** Multivariable Cox regression comparing the prognostic impact of differentially expressed genes in TNFα^+^ TAMs. HR, hazard ratios, 95% confidence intervals, and P values are shown. **E** Violin plot showing the signature gene expression of TNFα^+^ TAMs, split by the Bone Invasive states. **F**, **G** UMAP plot and Violin plot reflecting the significant enrichment of TNFα^+^ TAMs gene signature in BI-CX3CR1 + TREM2 + Mo-TAM. **H** Kaplan-Meier plot displaying the Progression Free Survival in patients with PitNETs, stratified by TNFα^+^ TAMs gene signature expression levels

We next employed multiplex immunofluorescence staining targeting CX3CR1, TREM2, CD68, and TNF-α to confirm the presence of TNF-α^+^TAMs in the BI sample (Fig. 3A, Figure S3 A). Co-expression of CX3CR1, TREM2, and CD68 is observed with nearby TNF-α expression in some areas, indicating the presentation of TNF-α^+^ TAM (Figure S3 B); in other areas, co-expression of CX3CR1, TREM2, and CD68 is observed without nearby TNF-α expression, indicating the presentation of TNF-α^−^ TAM (Figure S3 C). Given that our analysis revealed a higher prevalence of TNF-α^+^ TAMs in the BI group, we anticipate the BI group would also have higher TNF-α expression. To confirm this, we used quantitative PCR on tumor specimens, which showed elevated TNF-α mRNA levels in the BI group compared to Non-BI counterparts (Fig. 3B). In addition, enzyme-linked immunosorbent assay (ELISA) results showed higher TNF-α concentrations in the blood of patients with BI PitNETs relative to those with Non-BI PitNETs (Supplementary Figure S3D). To further validate the correlation between TNF-α^+^TAM presentation and TNF-α expression, we performed a deconvolution analysis that revealed a significant positive correlation between the proportion of TNF-α^+^TAMs and TNF-α expression levels (Figure S3E). We next sought to examine the clinical relevance of TNF-α activation in PitNETs. By stratified patients into two subgroups based on TNF-α expression levels from a cohort of bulk-sequenced PitNETs, we found that patients with high expression of TNF-α experienced a shorter progression-free survival (PFS) (Fig. 3C). Lastly, to establish a prognostic evaluation system, we defined a gene signature specific to TNF-α^+^ TAM and compared the prognostic impact of differentially expressed genes between BI and Non-BI groups using a multivariable COX regression (Fig. 3D). Our results indicate that the signature gene was mainly highly expressed in the TNF-α^+^ TAMs within the BI group (Fig. 3E), and the signature scores in the BI TNF-α^+^ TAMs were significantly higher than those in the Non-BI group (Fig. 3F, G). Survival analysis indicated a high TNF-α^+^ TAM signature is associated with poor prognosis. These findings suggest that this signature could be utilized to predict the prognosis of PitNET patients, offering valuable insights for clinical practice. (Fig. 3H).

### TNF-α+ TAMs promote osteoclast differentiation

**Fig. 4:**
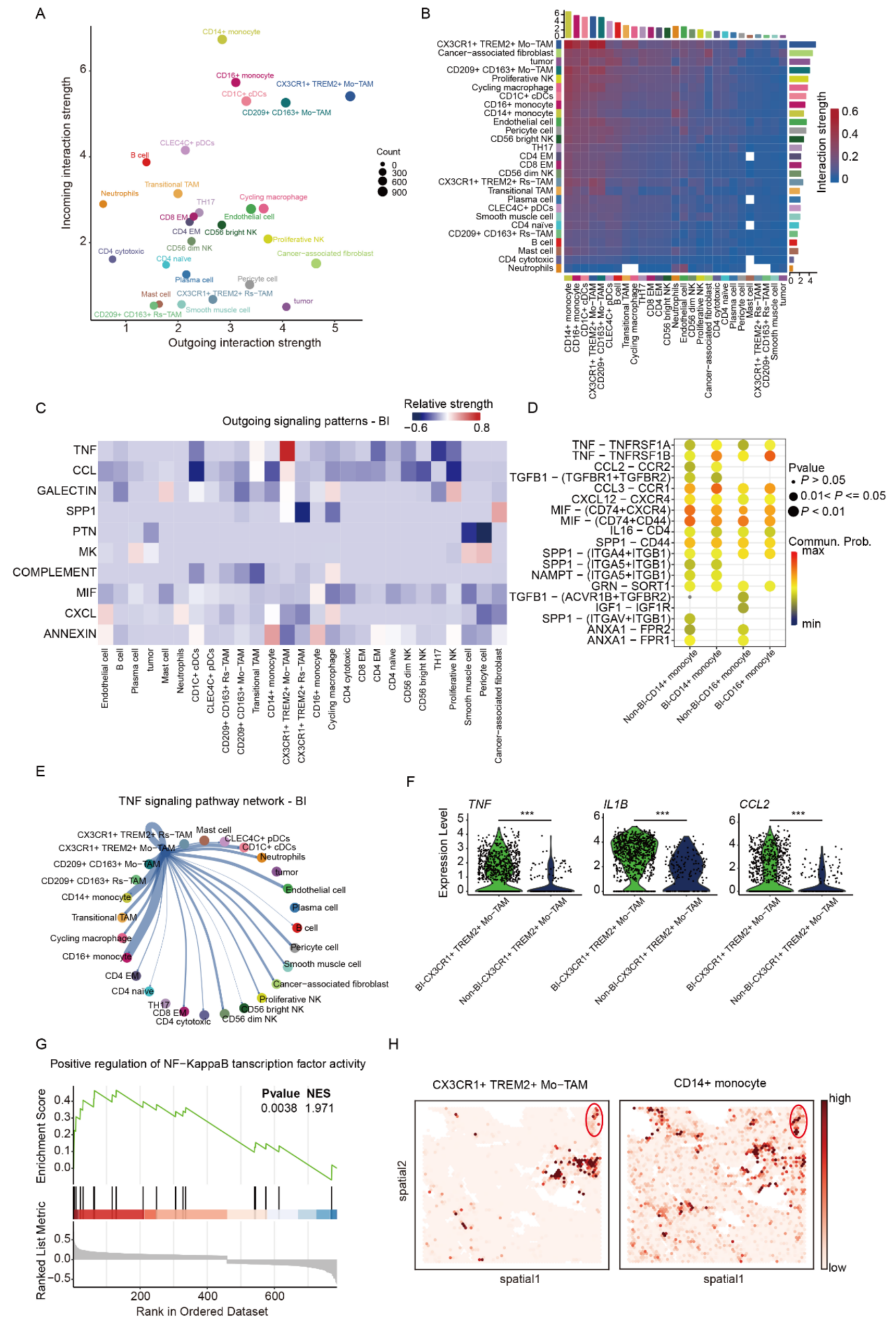
TNFα^+^ TAMs promote osteoclast differentiation. **A** Scatterplot showing the interaction strength of outgoing and incoming secretory signals in BI-PitNETs. **B** Heatmap of interaction strengths between different cell populations in BI-PitNETs. The top bar plot indicates the sum of incoming signals, and the right bar plot indicates the sum of outgoing signals. **C** Heatmap showing the relative strength of outgoing signaling pathways in BI-PitNETs. The red color indicates signaling pathways strengthened in BI compared to Non-BI. **D** Circos plots of the TNF signaling pathway network in BI-PitNETs. **E** Dot plot showing communication probability between top-ranking ligands expressed by TNFα^+^ TAMs and receptors on monocyte cells. **F** Violin plot showing the expression of top-ranking ligands in TNFα^+^ TAMs. **G** GSEA of differentially expressed genes ranked by log2FC between BI-CD14 + monocytes and Non-BI-CD14 + monocytes. NES, normalized enrichment score. **H** Spatial feature plots showing co-localization of TNFα^+^ TAMs (left) and CD14 + monocytes (right) in spatial transcriptomic deconvolution analyses. The bone-containing area is within the red elliptical region

To investigate the role of TNF-α^+^ TAMs in osteoclast differentiation, we conducted a cellular interaction analysis in the BI group (Supplementary Table S5). Our finding revealed that TNF-α^+^ TAMs act as principal signal transmitters, predominantly interacting with monocytes as receivers (Fig. 4, A, B). However, this interaction is markedly weakened (Figure S4, A, B) in the Non-BI group. This implies that TNF-α^+^ TAMs of BI PitNETs may influence monocytes through specific output signals, thereby promoting bone invasion. Furthermore, TNF-α^+^ TAMs in BI tumors exhibited enhanced TNF and CCL signaling output, with monocytes being the primary recipients of these signals (Fig. 4C; Figure S4C), indicating that TNF-α^+^ TAMs might affect monocytes through TNF-α. Previous studies have noted that TNF-α could promote monocyte differentiation to osteoclasts through NF-KB and MAPK signaling [2, 21, 22]. Additionally, the binding of CCL2 to CCR2 could enhance monocyte chemotaxis and migration and osteoclast formation [23]. In line with these findings, our receptor-ligand pair analysis identified the TNF-TNFRSFIA/B, CCL2-CCR2 as key communication channels between TNF-α^+^ TAMs and CD14^+^ monocytes (Fig. 4, D). In the TNF signaling pathway network of the BI group, TNF-α^+^ TAMs were centrally positioned and closely communicated with monocytes (Fig. 4E), a phenomenon was not observed in the Non-BI group (Figure S4D). Notably, CD14^+^ monocytes significantly overexpress receptor genes such as TNFRSF1A, TNFRSF1B, and CCR2, further confirming the interaction between TNF-α^+^ TAMs and CD14^+^ monocytes (Figure S4 E). Following this, we conducted a comparative analysis of the expression levels of genes such as *TNF*, *IL1B*, and *CCL2* between the BI and Non-BI groups. We found that TNF-α^+^ TAMs in the BI group exhibited significantly higher expression of these genes. Meanwhile, PCR results indicated high expression of *TNF* and *CCL2* in tumor samples (Fig. 3B; Figure S4 F), confirming this effect at the genetic level (Fig. 4F). We also performed GSEA analysis on known NF-KappaB pathways associated with bone invasion [24]. Our analysis revealed a significant upregulation of NF-KappaB transcription factor activity signaling pathways in CD14^+^ monocytes of the BI group compared to the Non-BI group (Fig. 4G). Most importantly, spatial transcriptome deconvolution showed that TNF-α + TAMs and CD14 + monocytes were both clustered around the bone (within the ellipse in Fig. 4H) and located in close proximity to each other (Fig. 4H), providing the possibility for cell-cell interactions. Finally, we analyzed the expression of genes related to the “Positive regulation of NF-KappaB transcription factor activity” pathway and found that CD14^+^ monocytes in the BI group were significantly overexpressed (Figure S4 G), indicating activation of NF-KappaB. This finding confirms the association between CD14^+^ monocytes and osteoclast differentiation in the BI group. To verify the presence of osteoclasts in PitNET samples, we validated the osteoclast markers *CTSK*, *ACP5*, and *MMP9* in the spatial transcriptomics data, confirming the existence of osteoclasts (Figure S4 H). In summary, this study found that TNF-α^+^ TAMs in the BI group can promote the differentiation of *CD14*^+^ monocytes into osteoclasts through the secretion of TNF-α, CCL2, and IL1B, thereby leading to bone destruction in BI PitNETs.

### Tumor-secreted IL34 induces high TNF expression in macrophages

**Fig. 5:**
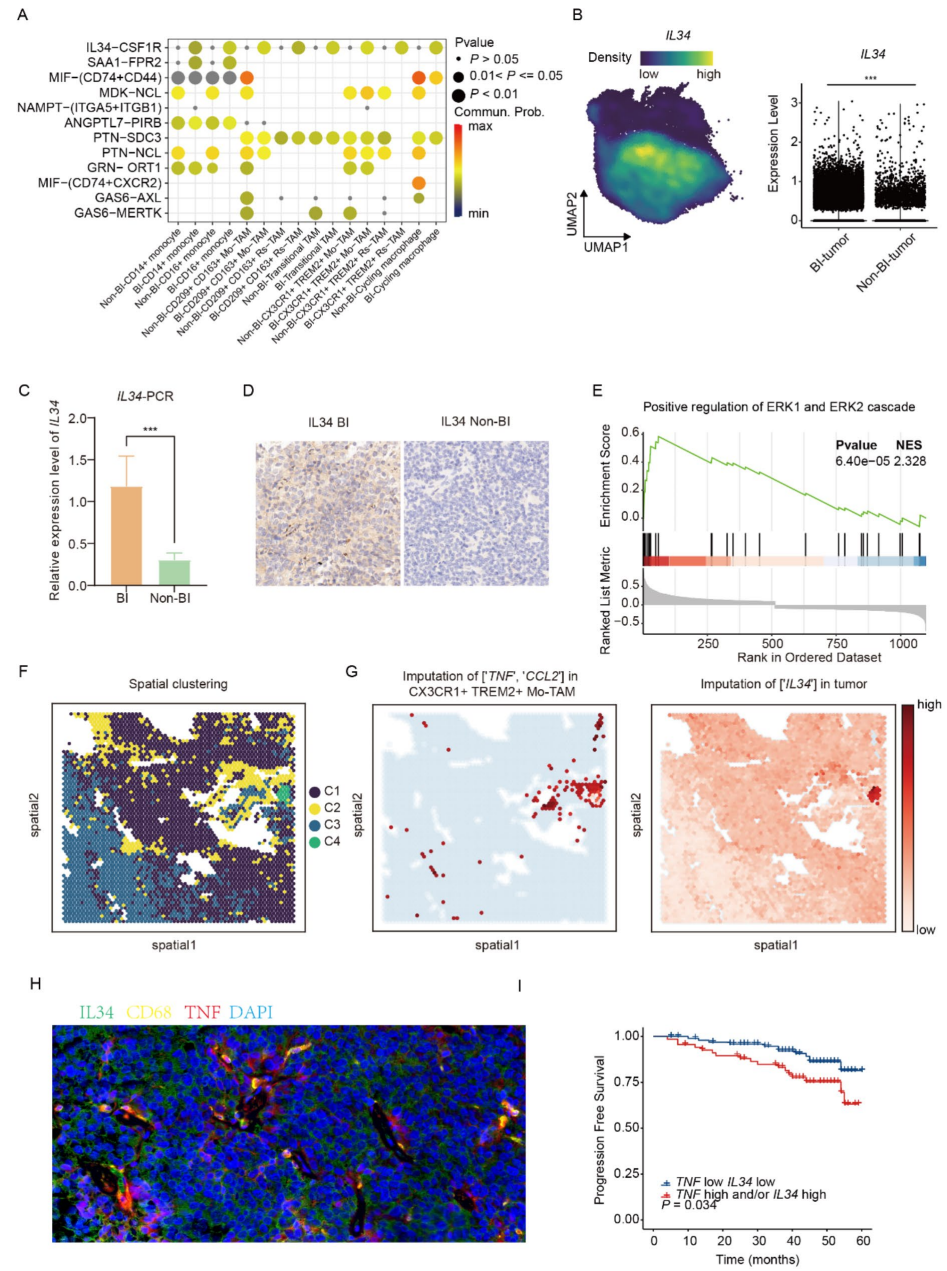
Tumor-derived IL34 promotes high *TNF* expression in macrophages. **A** Dot plot showing communication probability between top-ranking ligands expressed by tumor cells and receptors on subclusters of monocytes and macrophages. **B** UMAP and Violin plots revealing significantly high expression of IL34 in BI-PitNETs. ***, P value < 0.001. **C** PCR detected the relative expression levels of IL34 in 5 cases of BI and 5 cases of Non-BI PitNETs, with significantly higher IL-34 expression in the BI group. The H-scores for the BI and Non-BI groups are 208.05 ± 17.82 and 24.63 ± 5.58, respectively (*p* < 0.001). **D** Immunohistochemical staining was used to detect the relative expression levels of IL34 in 8 cases of BI and 8 cases of Non-BI PitNETs, showing significantly higher IL-34 expression in the BI group. **E** GSEA of differentially expressed genes ranked by log2FC between BI-TNFα^+^ TAMs and Non-BI-TNFα + TAMs. NES, normalized enrichment score. **F** Spatial feature plot demonstrating subcluster distribution following Kmeans clustering for spatially weighted PCA of tumor cells. **G** Spatial feature plot showing imputed expression values of *TNF*, *CCL2* in TNFα^+^ TAMs and, IL34 in tumor cells, as predicted by the trained single-cell data applied to spatial transcriptome space. **H** Immunofluorescence staining was performed to detect the expression of IL34 and TNF-α in BI PitNETs. IL34 (green) was widely expressed in the tumor tissue, while TNF-α (red) was mainly expressed around macrophages (yellow). **I** Kaplan-Meier plot displaying the Progression Free Survival in patients with PitNETs, stratified by expression levels of *TNF* and/or *IL34* at the first quartile cutoff point

Our subsequent investigation aimed to identify the potential regulator for TNF over-expression in TNF-α^+^ TAMs in the BI group. By comparing the communication probability between top-ranking ligands secreted by tumor cells and receptors on subclusters of monocytes and macrophages, we found that tumor cells predominately communicate with TNF-α^+^ TAMs via IL34-CSF1R in BI PitNETs (Fig. 5A). Meanwhile, we observed higher expression of IL34 in the BI group than in the Non-BI group, evidenced at both gene expression level and protein expression levels (Fig. 5, B, C, D). However, no intergroup differences were observed in IL34 levels in the blood (Figure S5 A), indicating that IL34 secretion by BI PitNETs is confined to the tumor microenvironment, where the excessively secreted IL-34 can act on TNF-α^+^ TAMs. In addition, it has been reported that production of TNF-α could be induced by ERK1/2 signaling activation [25]. To explore this, we performed GSEA analysis on the differentially expressed genes between BI-TNF-α^+^ TAMs and Non-BI-TNF-α^+^ TAMs. The results showed a significant upregulation of ERK1/2 signaling in BI-TNF-α^+^ TAMs (Fig. 5, E). Furthermore, genes involved in the positive regulation of ERK1 and ERK2 pathways were also significantly upregulated in BI-TNF-α^+^ TAMs. (Figure S5, B).

To further verify the relationship between IL34 and tumor cells, we localized tumor subgroups in the spatial transcriptome and found that the spatial location of the tumor with high IL34 expression was close to the TNF-α^+^ TAMs with high TNF and CCL2 expression (Fig. 5F, G; Figure S5 C, D). Additionally, deconvolution analysis of the BI group indicated a positive correlation between the predicted proportion of tumor cells and IL34 expression levels (Figure S5, E). These results support the hypothesis that tumor cells enhance TNF-α secretion from TNF-α^+^ TAMs through IL34. To validate this hypothesis, we performed multiplex immunofluorescence staining in tumor specimens and confirmed the proximal co-localization of IL34-positive tumor cells and TNF-α^+^ TAMs (Fig. 5, H). Finally, to explore the clinical significance of elevated IL34 and TNF-α, survival analysis further indicated that high expression of TNF and/or IL34 is associated with poor prognosis (Fig. 5, I).

### Landscape of T cells and fibroblasts in PitNETs

To determine the potential association of T cells infiltration and bone invasion in PitNETs, we re-clustered a total of 5,090 T cells into various subgroups, including CD4 naive, CD4 effector memory (EM), CD4 cytotoxic, TH17, CD8 EM, CD56 dim natural killer (NK), CD56 bright NK, and proliferative NK cells (Figure S6 A). The expression of cell-type-specific markers in each cell cluster is detailed in Figure S6 B and C. In the BI group, there was an increased proportion of CD4 naive and TH17 cells, accompanied by a decrease in CD56 dim NK cells, as depicted in Figure S6 D. Notably, the elevated TNF-α expression in TH17 cells compared to other T cell subsets suggests a potential role of TH17 cells in bone invasion. However, no significant difference in TNF-α expression between the BI and Non-BI groups was observed. (Figure S6 E). Furthermore, differential gene expression analysis between TH17 cells and other T cell types highlighted an enrichment in immune-related pathways, including Cytokine Signaling in the Immune System and the Positive Regulation of Osteoclast Differentiation (Figure S6 F). Given that TH17 cells can promote bone resorption in periodontitis [26], our study also found that TH17 cells highly express TNF-α, and the differential genes between TH17 and other T cells are enriched in Positive Regulation of Osteoclast Differentiation, indicating that our findings are consistent with the existing literature. Additionally, the proliferation of TH17 cells is known to be stimulated by IL23^27^, and we observed high IL23R expression in our TH17 cells (Figure S6 C), suggesting they are actively proliferating and potentially in a functionally active state. However, no significant difference in the proportion of TH17 cells or the TNF-α secretion level between the BI and Non-BI groups was noted, suggesting that TH17 cells may be responsible for bone resorption under normal physiological conditions. RNA velocity analysis indicated that the differentiation of TH17 cells is likely derived from CD4 naive cells (Figure S6 G H).

Fibroblasts also play an important role in tumor proliferation, and invasion. In order to elucidate their role in bone invasion of PitNETs, we conducted a re-clustering analysis of fibroblasts. 545 fibroblasts were assessed (Figure S7 A and B) and classified into smooth muscle cells, pericytes, and cancer-associated fibroblasts (CAFs) based on cell marker gene expression (Figure S7 C). The BI group demonstrated an increased prevalence of CAFs and smooth muscle cells, with a concurrent decrease in pericyte abundance (Figure S7 D). The higher proportion of CAF in the BI group suggested its potential involvement in bone invasion. To study the differentiation and functional state of CAF, we performed pseudotime analysis, which revealed that CAF is in the late stage of differentiation. This high differentiation state indicates that CAF is functionally active (Figure S7 E). Further enrichment analysis of differential genes between CAF and other fibroblasts showed significant enrichment in pathways related to extracellular matrix formation and angiogenesis (Figure S7 F). However, there was no significant difference in the expression levels of these pathway-related genes between the BI and Non-BI groups (Figure S7 G, H). These results suggest that the immune microenvironment of BI PitNETs contains higher levels of CAF, which may contribute to bone invasion by promoting extracellular matrix formation and angiogenesis.

## Discussion

Bone-invasive PitNETs pose significant clinical challenges for neurosurgeons due to limited effective treatments. Evidence shows that the immune microenvironment plays a pivotal role in tumor development, including proliferation, invasion, and metastasis [4, 28, 29]. Targeting the immune microenvironment has emerged as a hot topic in cancer therapy and has shown promising initial results [30].

In this study, we uncovered the characteristics of macrophages in PitNETs and identified CX3CR1 + TREM2 + TAM1, which express high levels of TNF-α and IL1B. These cytokines are known to facilitate bone invasion by promoting the differentiation of monocytes into osteoclasts [2, 22]. Interestingly, the proportion of *CX3CR1*^+^*TREM2*^+^ TAM1 (TNF-α^+^ TAMs) was higher in the BI group than in the Non-BI group. Further analysis revealed that tumor cells in the BI group secrete excessive IL34, which, through the IL34-CSF1R interaction, activates the ERK1/2 signaling pathway in TNF-α^+^ TAMs, subsequently promoting TNF-α secretion. Cell-cell communication analysis and receptor-ligand pair analysis suggested that TNF-α^+^ TAMs may act on CD14^+^ monocytes through the TNF-TNFRSF1A/B and CCL2-CCR2 pathways, activating the NF-KB signaling pathway and promoting osteoclasts differentiation.

In summary, we identified a novel macrophage subset (TNF-α^+^ TAM) in PitNETs, derived from circulating monocytes with increased infiltration in BI PitNETs. These macrophages, characterized by *CX3CR1* and *TREM2* expression, significantly upregulate TNF-α and IL-1B, exacerbating bone invasion. Overall, this study highlights the crucial roles of cell-cell interactions in the process of bone invasion in PitNETs. By targeting key molecules on our identified axis of cell communications, pharmacologic interventions may exist to interrupt cellular interactions and cytokine secretion, thereby alleviating bone destruction. Thus, these findings offer new insights into the treatment strategy fo*r* bone-destructive PitNETs.

Colony-stimulating factor 1 receptor (CSF1R) is a conserved tyrosine kinase transmembrane receptor central to CSF1R signaling transduction [31, 32]. Mutations in CSF1R can impair osteoclast function, resulting in osteopetrosis [33, 34], which is consistent with our research findings. Additionally, CSF1R plays a significant role in the chemotaxis and accumulation of tumor-associated macrophages [35]. Therefore, blocking IL34-CSF1R signaling holds promise for improving bone invasion in PitNETs. CX3CR1, a cell surface protein primarily expressed in monocytes and macrophages, varies expression among different cell subtypes [36, 37]. The CX3CR1-CX3CL1 axis is critical in the immune microenvironment, as it has been shown to lead to infiltration of NK cells, monocytes, T cells into tumors [38], thereby altering the immune milieu and modulating immune responses [39, 40]. Studies have found that *CX3CR1*-deficient mice exhibit a reduction in microglia numbers during postnatal development, suggesting CX3CL1-CX3CR1 signaling may serve as a chemoattractant to facilitate the microglia aggregation in the brain [41].In breast cancer research, scholars injected MDA-231 cells into the bloodstream of CX3CL1 knockout mice, resulting in a 70% reduction in bone metastatic foci compared to wild-type mice. TREM2, a member of the triggering receptor expressed on myeloid cells family [42], is integral to the regulation of the immune microenvironment. In a study involving adipose tissue, researchers found that TREM2 drives the reshaping of the adipose tissue immune microenvironment, recruiting circulating monocytes to the vicinity of adipocytes, where they differentiate into TREM2^+^ lipid-associated macrophages. The absence of TREM2 eliminates the aggregation of macrophages around adipocytes [43]. Additionally, studies have demonstrated that in the alveolar bone of individuals with chronic periodontitis, there is a significant upregulation of *TREM2* expression. In a mouse model of periodontitis, knockout of *TREM2* results in reduced bone invasion [44]. The studies above highlight the dual role of CX3CR1 and TREM2 expression: they not only induce circulating monocytes to TME but also elicit invasive behavior of tumors. Targeting the IL34-CSF1R axis, the CX3CR1-CX3CL1 axis, and TREM2 may help reduce bone invasion in PitNETs. Further research is needed to validate this in the future.

## Conclusion

In this study, we elucidate the changes in the tumor microenvironment during bone invasion and identify the critical role of newly defined TNF-α + TAMs in promoting bone invasion of PitNETs. We also define the gene signature of TNF-α + TAMs, which can effectively predict the prognosis of BI PitNETs patients. This study lays a foundation for developing new molecular markers or therapeutic agents targeting BI PitNETs. More importantly, our findings provide new insights for research and treatment of other bone-invasive diseases.

## Electronic supplementary material

Below is the link to the electronic supplementary material.

Supplementary Material 1: Supplementary Figure S1: Basic information of the single-cell RNA-seq data. Supplementary Figure S2: Cluster characterization of myeloid cells. Supplementary Figure S3: CX3CR1+ TREM2+ Mo-TAM is the source of TNF in BI-PitNETs. Supplementary Figure S4: Cell–cell communication between TNFα^+^ TAMs and CD14^+^ monocytes. Supplementary Figure S5: Spatial distribution of IL34+ tumor.  Supplementary Figure S6: Characteristics of T cells in PitNET.  Supplementary Figure S7: Landscape of fibroblasts in PitNET.

Supplementary Material 2: Supplementary Table S1: Clinical information of patients from scRNA-seq. Supplementary Table S2: Analysis of the Correlation Between Pathological Types and Bone Invasion. Supplementary Table S3: PCR primers of mRNAs used for qRT-PCR. Supplementary Table S4: Differently expressed genes between Mo and Rs-derived CX3CR1 + TREM2 + TAM. Supplementary Table S5: Communication probability between ligands and receptors.

## Data Availability

The raw sequence data reported in this paper have been deposited in the Genome Sequence Archive (Genomics, Proteomics & Bioinformatics 2021) in National Genomics Data Center (Nucleic Acids Res 2022), China National Center for Bioinformation / Beijing Institute of Genomics, Chinese Academy of Sciences (GSA-Human: HRA008285) that are publicly accessible at https://ngdc.cncb.ac.cn/gsa-human.
