# Supplementary material for: Single-cell transcriptomics identify a novel macrophage population associated with bone invasion in pituitary neuroendocrine tumors
Source: J Exp Clin Cancer Res. 2025 Jan 27;44:27. doi: 10.1186/s13046-025-03296-9 (PMC11770939; doi:10.1186/s13046-025-03296-9)
Supplement: Supplementary file 1 — Supplementary Material 1: Supplementary Figure S1: Basic information of the single-cell RNA-seq data. Supplementary Figure S2: Cluster characterization of myeloid cells. Supplementary Figure S3: CX3CR1+ TREM2+ Mo-TAM is the source of TNF in BI-PitNETs. Supplementary Figure S4: Cell–cell communication between TNFα+ TAMs and CD14+ monocytes. Supplementary Figure S5: Spatial distribution of IL34+ tumor. Supplementary Figure S6: Characteristics of T cells in PitNET. Supplementary Figure S7: Landscape of fibroblasts in PitNET. [file 13046_2025_3296_MOESM1_ESM.docx]

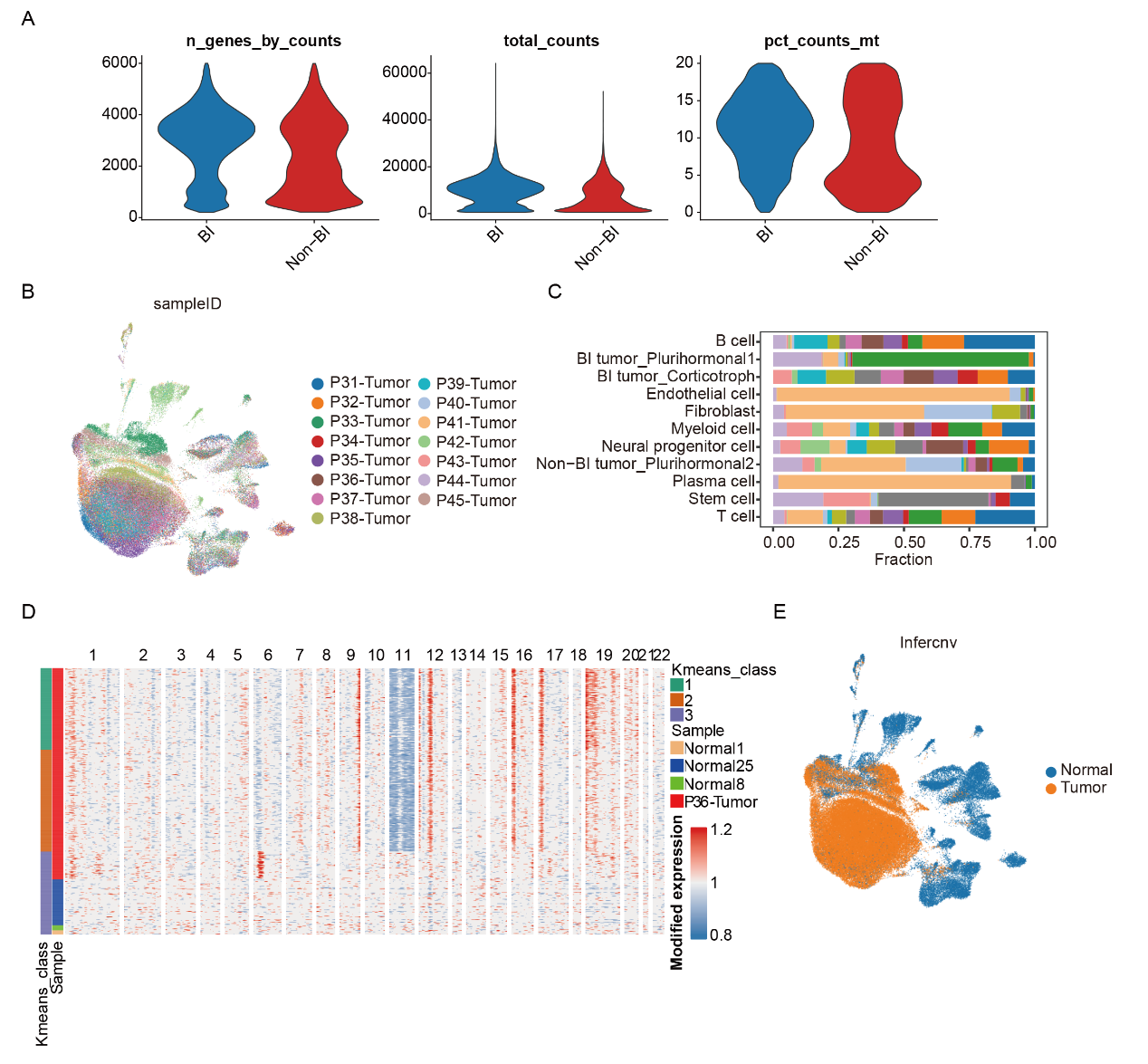


**Figure S1 Basic information of the single-cell RNA-seq data**

A Violin plot showing the number of expressed genes, UMI counts, and the percentage of mitochondrial genes within single-cell transcriptomic data for both BI and Non-BI samples.

B UMAP plot colored by sample ID.

C Bar plots displaying the proportion of each cell type across different sample IDs.

D Heatmap showing inferred copy number variations (CNVs) for cells from patient P36. Red highlights indicate amplifications, while blue highlights indicate deletions.

E UMAP plot classifying Normal and Tumor cells based on inferred CNV results.


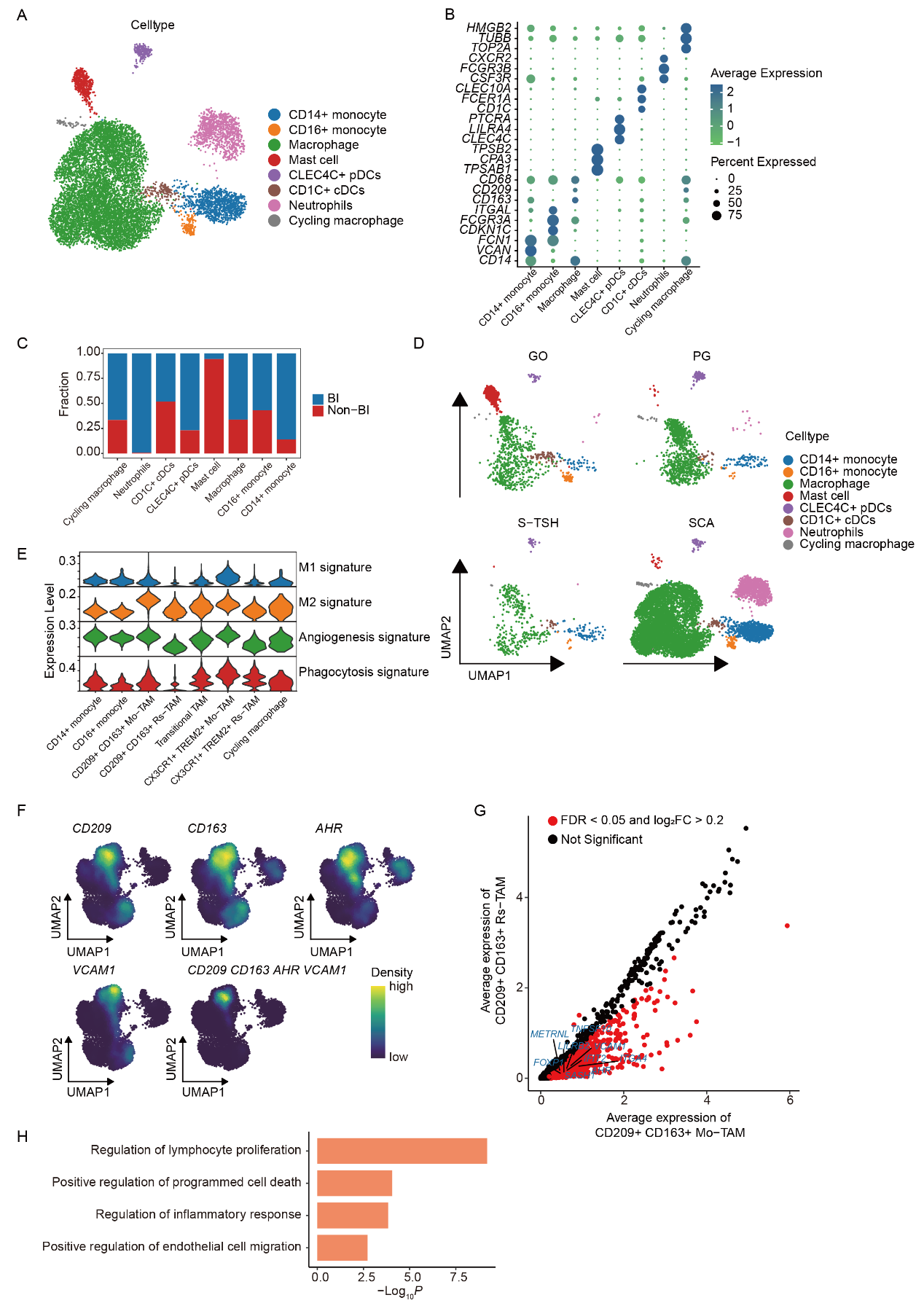


**Figure S2 Cluster characterization of myeloid cells.**

A UMAP plot of 10,346 myeloid cells colored by assigned cell type.

B Dot plot depicting the average expression of cell-type-specific markers in each cell cluster. The dot size indicates the proportion of cells expressing the marker genes in each cluster.

C Bar plot displaying the proportion of each cell type across Bone Invasive states.

D UMAP plots showing the cell composition of different pathological subtypes.

E Violin plot illustrating the expression of common M1, M2, Angiogenesis, and Phagocytosis signatures in each cluster, calculated using Ucell.

F UMAP plots showing the expression level of selected marker genes in CD209+ CD163+ TAMs.

G, H Differentially expressed genes and differentially activated pathways between CD209+ CD163+ Mo-TAMs and CD209+ CD163+ Rs-TAMs.


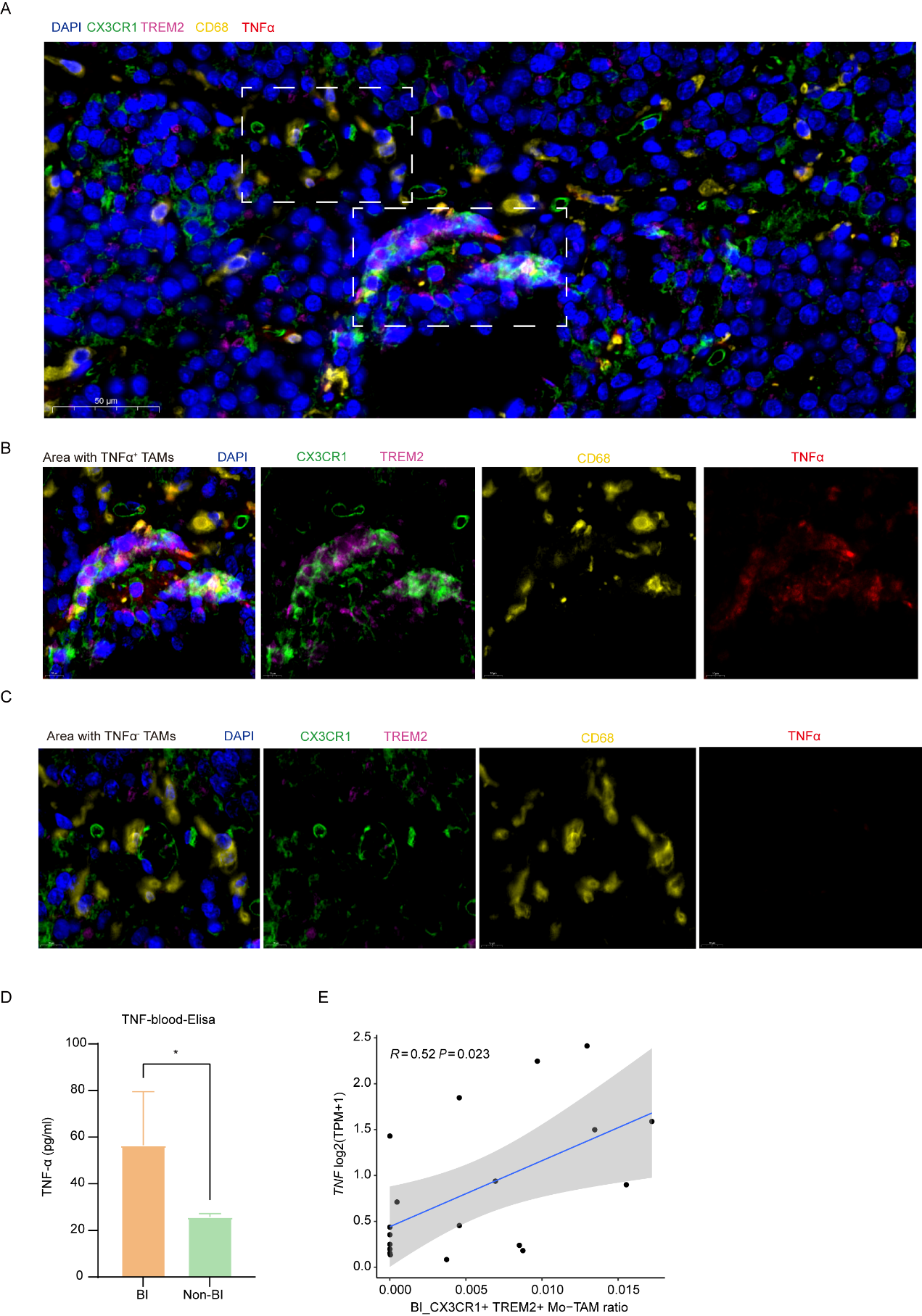
 **Figure S3 CX3CR1+ TREM2+ Mo-TAM is the source of TNF in BI-PitNETs.**

A Multiple immunofluorescence staining showing CX3CR1 (green), TREM2 (pink), CD68 (yellow), TNF-α (red), DAPI (blue), etc. in BI PitNET;

B Abundant TNF-α expression observed around macrophages co-expressing CX3CR1, TREM2, and CD68.

C Areas with TNF-α^-^ macrophages showing no TNF-α expression around CD68.

D ELISA detected the relative expression levels of TNF-α in the blood of patients with BI and Non-BI PitNETs (8 cases of BI and 8 cases of Non-BI PitNETs), showing significantly higher TNF-α expression in the BI group.

E Scatter plots demonstrating the correlation between estimated BI-CX3CR1+ TREM2+ Mo-TAM proportions and *TNF* expression in patients with bone invasion. Correlation coefficients (R) and p-values were calculated using the Spearman’s correlation test.


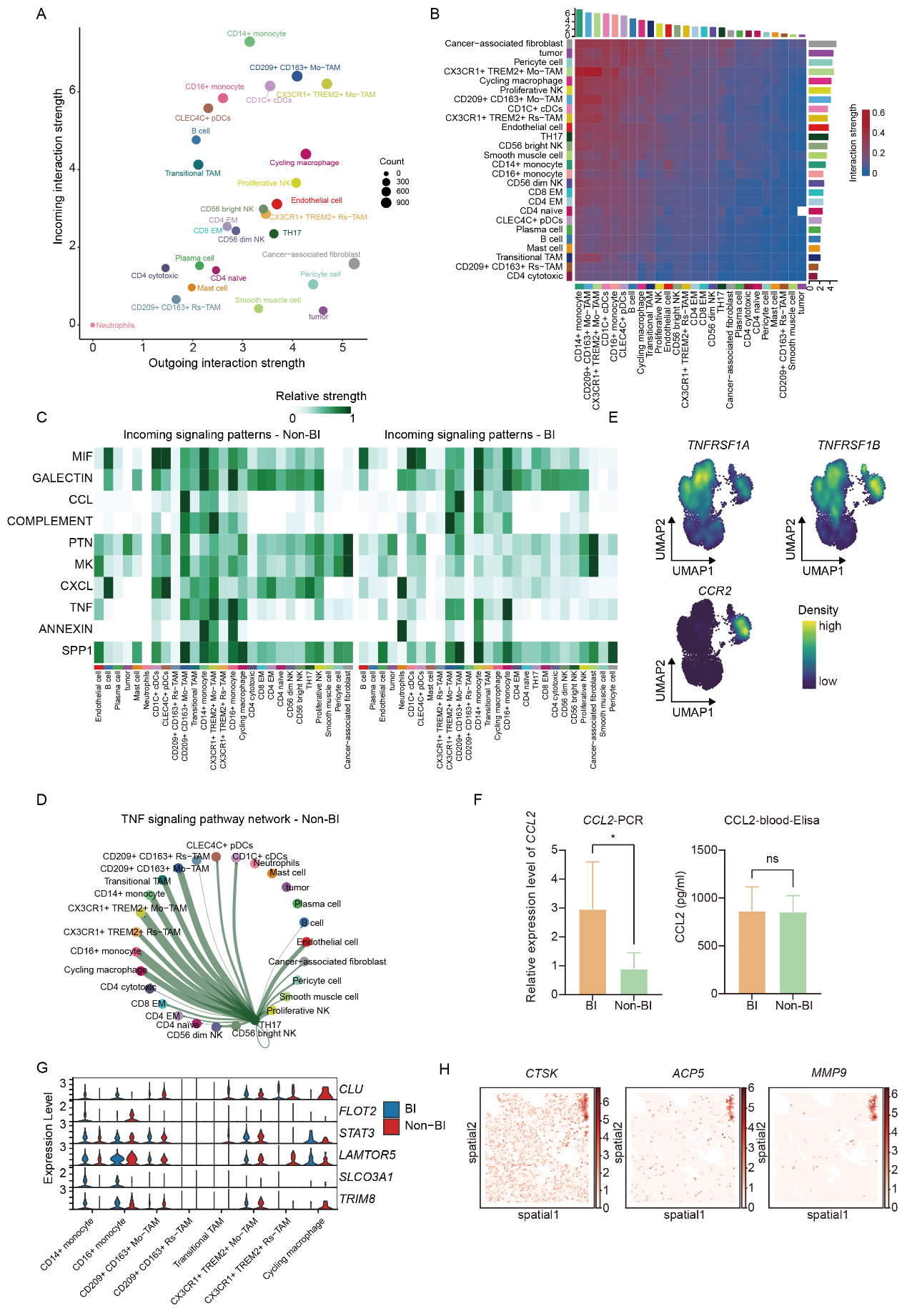


**Figure S4 Cell–cell communication between TNFα^+^ TAMs and CD14+ monocytes.**

A Scatterplot showing the interaction strength of outgoing and incoming secretory signals in Non-BI-PitNETs.

B Heatmap of interaction strengths between different cell populations in Non-BI-PitNETs. The top bar plot indicates the sum of incoming signals, and the right bar plot indicates the sum of outgoing signals.

C Heatmap comparing the relative strength of incoming signaling pathways between BI-PitNETs and Non-BI-PitNETs.

D Circos plots of the TNF signaling pathway network in Non-BI-PitNETs.

E Density plot visualizing the expression of receptors in CD14+ monocytes.

F PCR analysis detecting the expression of CCL2 in specimens from 8 cases of BI and 8 cases of Non-BI PitNETs (left), with significantly higher expression in the BI group. ELISA detected the relative expression levels of CCL2 in the blood of patients with BI and Non-BI PitNETs (8 cases of BI and 8 cases of Non-BI PitNETs), revealing no significant difference.

G Violin plot dictating the expression of selected genes involved in GO:0051092, positive regulation of NF−KappaB transcription factor activity.

H Spatial feature plots showing osteoclast-specific marker gene expression levels in spatial transcriptomic analyses.


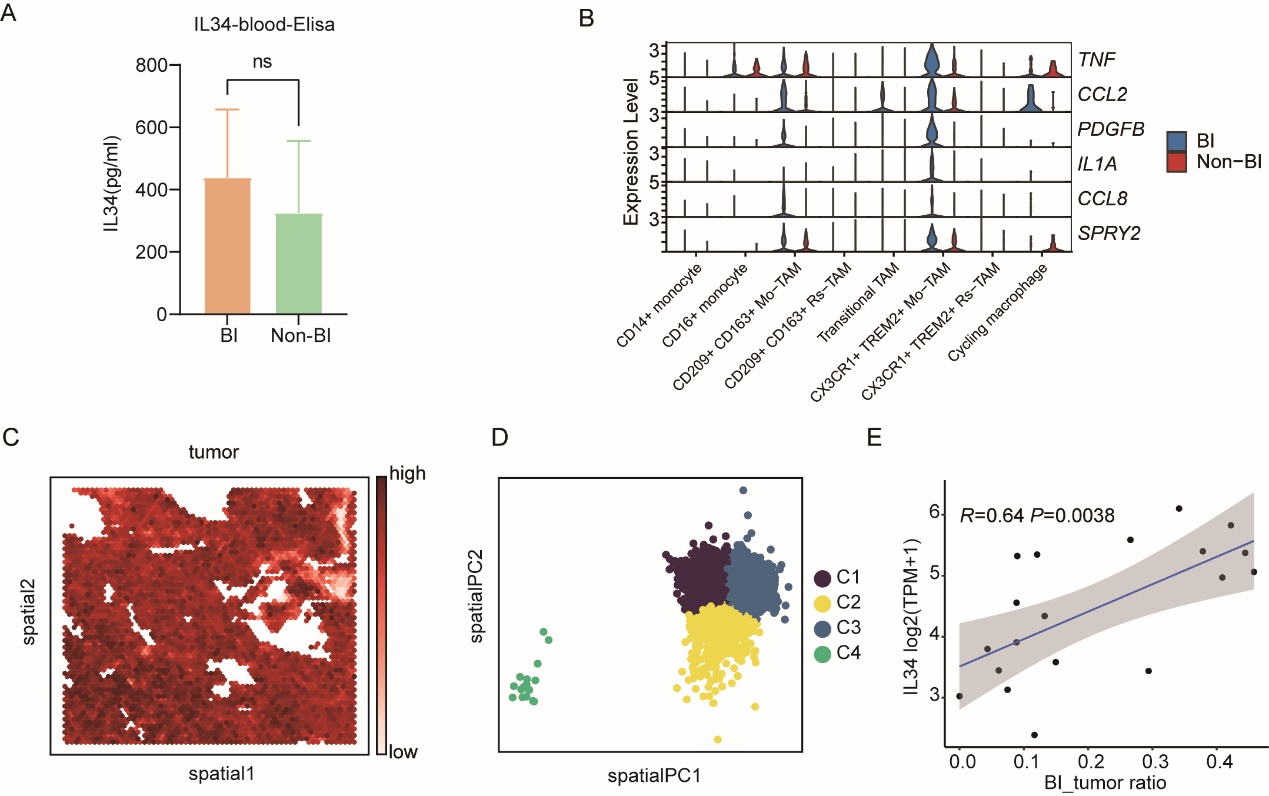


**Figure S5 Spatial distribution of IL34+ tumor.**

A ELISA detected the relative expression levels of IL34 in the blood of patients with 8 cases of BI and 8 cases of Non-BI PitNETs, with no significant difference.

B Violin plot showing the expression of selected genes involved in GO:0070374, positive regulation of ERK1 and ERK2 cascade.

C Spatial feature plots showing the localization of tumor cells in spatial transcriptomic deconvolution analyses.

D PCA plot showing Kmeans clustering for spatially weighted PCA of tumor cells.

E Scatter plots demonstrating the correlation between estimated BI-tumor proportions and *IL34* expression in patients with bone invasion. Correlation coefficients (R) and p-values calculated using the Spearman’s correlation test.


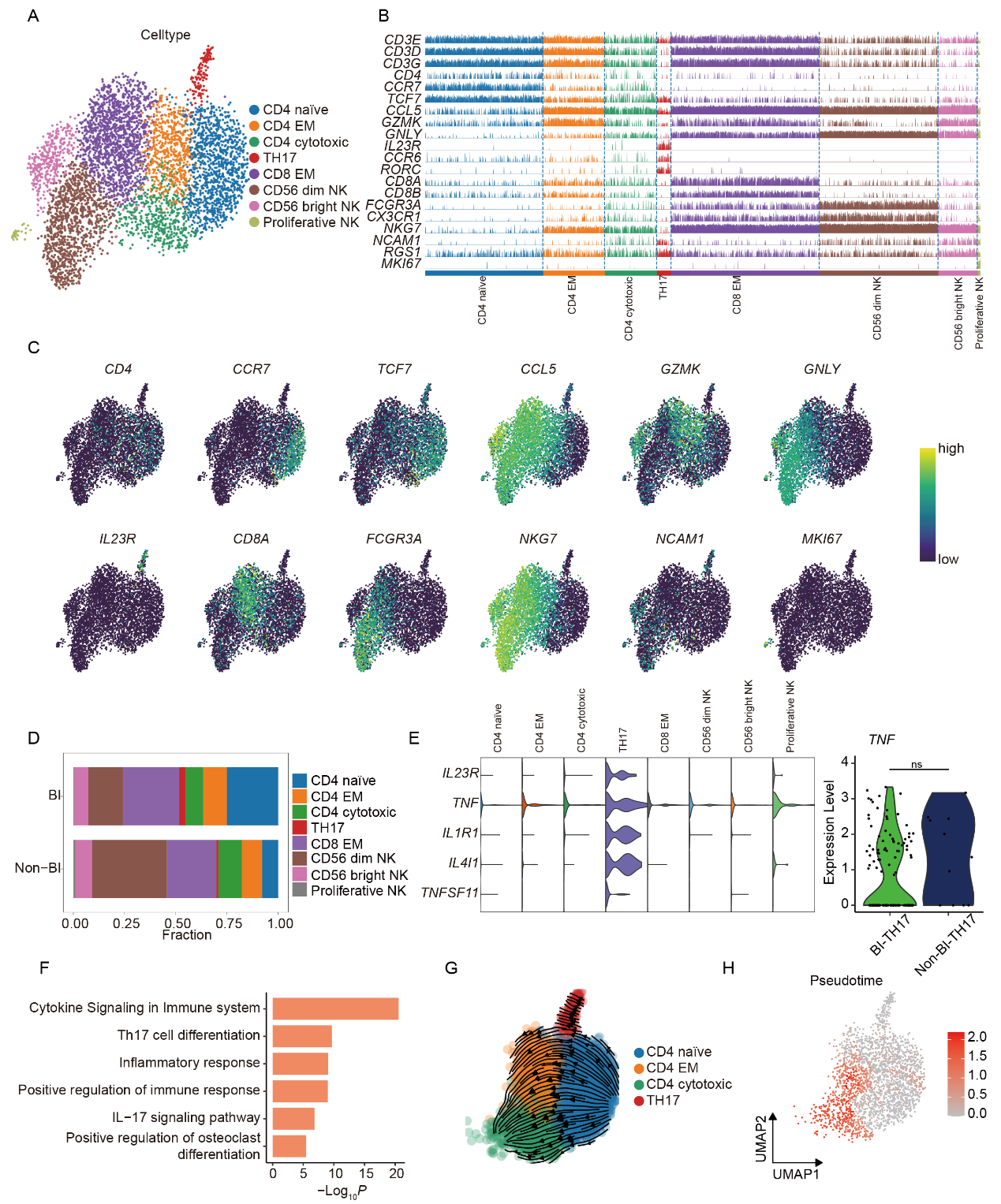


**Figure S6 Characteristics of T cells in** PitNET**.**

A UMAP plot of 5,090 T cells, colored by assigned cell type.

B Track plot illustrating the expression of cell-type-specific markers across each cell cluster.

C UMAP plots showing expression levels of selected marker genes.

D Bar plot displaying the proportion of each cell type across Bone Invasive.

E Violin plot showing the expression of selected genes in TH17.

F Bar plot depicting pathways enriched in genes with higher expression in TH17 compared to other T cell types.

G, H Inferred developmental trajectory of CD4+ T cells by RNA velocity (G) and monocle3 (H) on UMAP. Colors indicate cell type (left) or pseudotime (right).


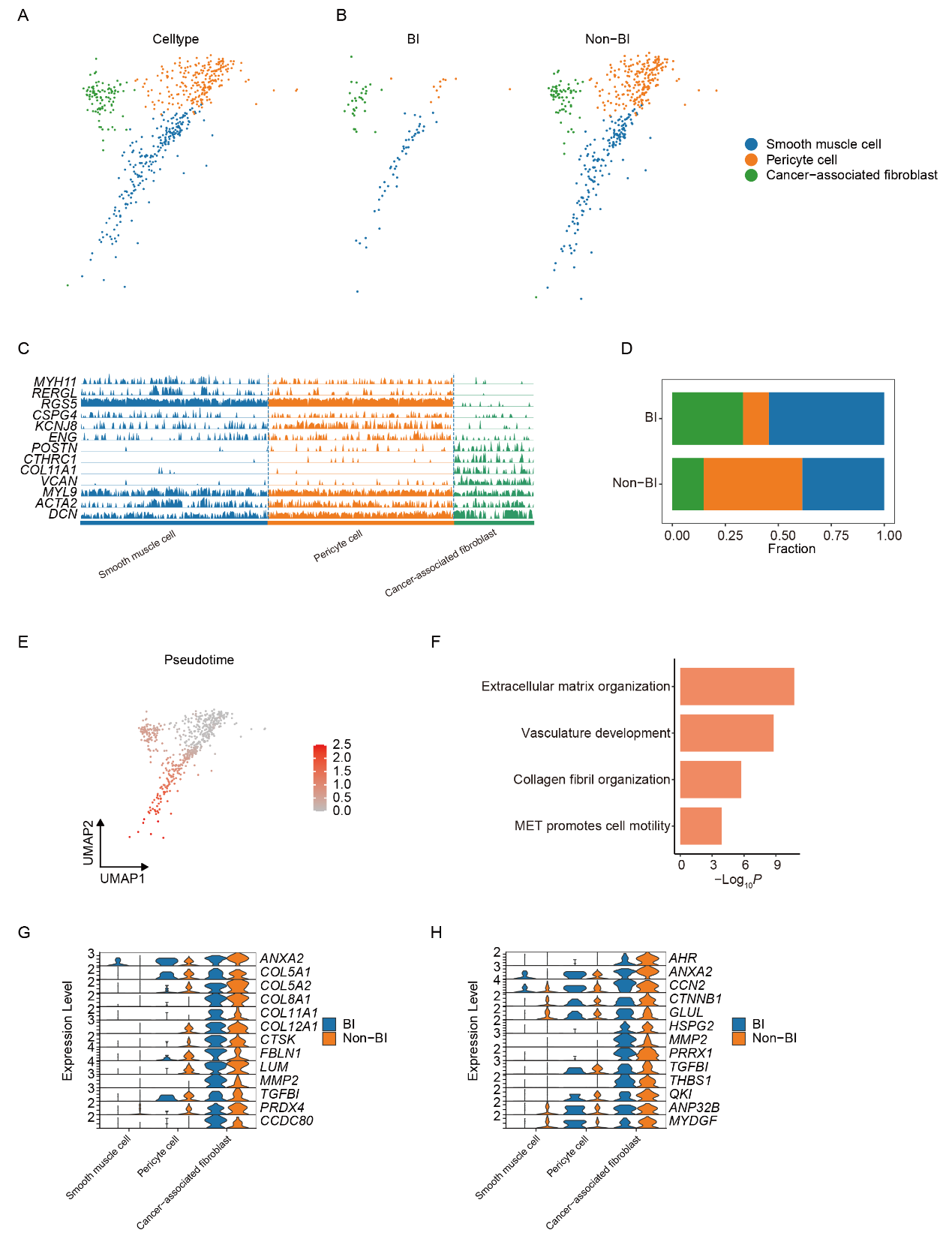


**Figure S7 Landscape of fibroblasts in** PitNET**.**

A UMAP of 545 fibroblasts, colored by assigned cell type.

B UMAP plots showing the distinct cell composition of BI and Non-BI samples.

C Track plot showing the expression of cell-type-specific markers across each cell cluster.

D Bar plot displaying the proportion of each cell type across Bone Invasive states.

E Inferred developmental trajectory of fibroblast cells using monocle3 on UMAP, with colors indicating pseudotime.

F Bar plot depicting pathways enriched in genes with higher expression in Cancer−associated fibroblasts compared to other fibroblasts.

G, H Violin plot showing the expression of selected genes involved in GO:0030198, extracellular matrix organization (G), and GO:0001944, vasculature development (H).
